# Genome-Based Prediction of Bacterial Antibiotic Resistance

**DOI:** 10.1128/JCM.01405-18

**Published:** 2019-02-27

**Authors:** Michelle Su, Sarah W. Satola, Timothy D. Read

**Affiliations:** a Department of Infectious Diseases, Emory University, Atlanta, Georgia, USA; b Antimicrobial Resistance and Therapeutic Discovery Training Program, Emory University, Atlanta, Georgia, USA; c Antibiotic Resistance Center, Emory University, Atlanta, Georgia, USA; d Emory Investigational Clinical Microbiology Laboratory, Emory University, Atlanta, Georgia, USA; Boston Children’s Hospital

**Keywords:** antibiotic resistance, genome-based prediction

## Abstract

Clinical microbiology has long relied on growing bacteria in culture to determine antimicrobial susceptibility profiles, but the use of whole-genome sequencing for antibiotic susceptibility testing (WGS-AST) is now a powerful alternative. This review discusses the technologies that made this possible and presents results from recent studies to predict resistance based on genome sequences. We examine differences between calling antibiotic resistance profiles by the simple presence or absence of previously known genes and single-nucleotide polymorphisms (SNPs) against approaches that deploy machine learning and statistical models. Often, the limitations to genome-based prediction arise from limitations of accuracy of culture-based AST in addition to an incomplete knowledge of the genetic basis of resistance. However, we need to maintain phenotypic testing even as genome-based prediction becomes more widespread to ensure that the results do not diverge over time. We argue that standardization of WGS-AST by challenge with consistently phenotyped strain sets of defined genetic diversity is necessary to compare the efficacy of methods of prediction of antibiotic resistance based on genome sequences.

## Abstract (precis)

Clinical microbiology has long relied on growing bacteria in culture to determine antimicrobial susceptibility profiles, but the use of whole-genome sequencing for antibiotic susceptibility testing (WGS-AST) is now a powerful alternative. This review discusses the technologies that made this possible and presents results from recent studies to predict resistance based on genome sequences.

## IMPORTANCE OF AST

Antibiotic-resistant bacterial infections are a global threat. Many previously manageable bacterial infections are becoming increasingly hard to treat. The CDC recently estimated that in the United States alone, at least two million people will be infected by a drug-resistant bacterium each year, and at least 23,000 people will die as a result (1). Rising rates of resistance amplify the morbidity and economic burden associated with infections. Even successful treatment can come with increased complications, as more-toxic drugs of last resort, like colistin, are being used more frequently because bacteria are not susceptible to less-toxic antibiotics. For the management of infections in both the clinic and community, accurate detection of antimicrobial resistance is necessary to guide treatment decisions.

Culture-based antimicrobial susceptibility testing (AST) is still the primary method employed by clinical laboratories. While there are other promising approaches for phenotypic detection and rapid nonsequencing genetic methods currently in use (e.g., PCR for resistance determinants) (2), dramatic progress over the past 5 years in the applications of genomics has caught the attention of the clinical microbiology community. Whole-genome sequencing for antimicrobial susceptibility testing (WGS-AST) offers the potential to provide rapid, consistent, and accurate predictions of every known resistance phenotype for a strain, as well as simultaneously provide rich surveillance data. Recent reviews of the subject have focused on clinical standardization (2–4). Here, we concentrate on the problem of predicting resistance based only on genome sequence and consider the future symbiotic relationship between genomics and phenotypic-based AST.

## POTENTIAL ADVANTAGES OF WGS-AST

**FIG 1:**
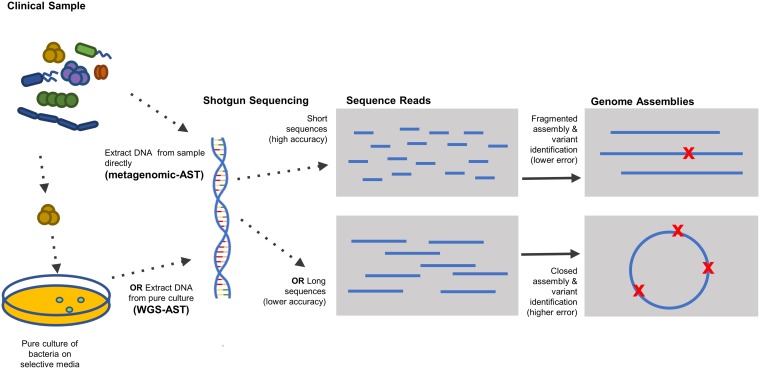
Overview of genome sequencing and how it is used in WGS-AST. DNA is extracted from directly from bacteria in clinical samples (metagenomics) or more commonly, from cultured bacterial colonies. Sequencing technologies fragment the DNA and then randomly sequence to produce a library of reads (stored in FASTQ files). The reads are assembled into genomic scaffolds *in silico*. Sequencing is performed either using short-read second-generation technology, which tends to produce fragmented whole-genome assemblies of high accuracy, or long-read third-generation technologies that have higher error rates but more complete assemblies. WGS-AST algorithms operate on the raw reads and/or assembled contigs.

WGS-AST follows selective culturing of the bacterium of interest from a clinical sample (Fig. 1). AST following direct shotgun sequencing of clinical samples (metagenomic-AST) is also possible (see, e.g., reference 5) and is the subject of intense current research, but it is more complex, expensive, and prone to false-negative results due to the potentially low abundance of the pathogen of interest relative to host DNA. Metagenomic-AST also includes approaches that enrich a library of antibiotic resistance DNA fragments from complex clinical samples before sequencing (6). Slow-growing or hard-to-culture bacteria (such as Mycobacterium tuberculosis [6, 7]) are important early targets for metagenomic-AST because DNA sequencing may be easier and faster than obtaining enough culture growth for phenotypic testing. This review will focus on the WGS-AST application, but many principles may also apply to other sequence-based AST approaches. Compared to culture-based AST or nucleic acid amplification tests (NAATs), which are often limited by the number of resistant phenotypes that can be determined from one test, WGS-AST can ascertain the antibiotic resistance phenotypes of the entire genome simultaneously, and phenotypes where multiple loci contribute can be easily screened (instead of performing multiplex PCRs). Once collected, the genome sequence data are stored digitally and can be queried for other purposes (e.g., complete-genome multilocus sequence typing [cgMLST] genotype [8] and virulence [9]). Genomes can be sequenced to very high levels of depth, giving very accurate sequence data. Unlike NAATs, there is no reliance on primer specificity for template amplification, reducing the possibility of false-negative results. The accumulation of genomes in clinical laboratories creates a data source that can be used to survey the evolution of pathogens (10). If new antibiotic resistance loci are discovered, these databases can be immediately scanned to understand how long these genes have been circulating and how they may have entered the clinical setting. One of the first examples of this type of retrospective surveillance investigation was performed for the emergence of *mcr-1* colistin resistance in Germany (11, 12), where a database of 577 Enterobacteriaceae genomes from animals and humans was searched to find four previously undiagnosed colistin-resistant isolates.

## NEXT-GENERATION SEQUENCING TECHNOLOGIES DRIVING WGS-AST

Two waves of sequencing technology innovation, the “second generation” starting in the mid-2000s and the “third generation” from about 2010 on, transformed all aspects of genomics and set the stage for WGS-AST (13, 14) (Table 1 and Fig. 1). Second-generation instruments, represented by the currently dominant Illumina sequencing-by-synthesis technology, sharply reduced the cost to generate data (i.e., $/Mb), which led to large-scale sequencing of thousands of pathogen genomes and the use of shotgun metagenomics for clinical diagnostics. Illumina sequence reads are short (≤300 bp) (15), typically paired-end, and have low per-base error rates (typically <0.1%). Illumina sequencing allows deep shotgun coverage with high consensus accuracy, but *de novo* assembly typically results in genomes fragmented into multiple contigs and collapsed repeat regions (Fig. 1).

**TABLE 1:** Genomics terms

Term	Definition^*a*^
Quality score	A measure of the probability of an inaccurate base call, typically represented by the Phred score [Q = −10 log_10_(P)] (113). A Q of 10 represents a 1 in 10 chance of error, whereas a Q of 40 is a 1 in 10,000 chance of error.
Coverage	A measure of how many instances a base was sequenced, as quantified by number of unique reads mapped to that position in the genome. A “genome of coverage of 30×” means that on average, each base of the genome has 30 reads mapped.
Sequence read	Inferred nucleotide sequence of a genome fragment. Reads range from short (≤300 bp) to long (5 to 100+ kbp).
Contig	A contiguous sequence created by assembling multiple overlapping sequence reads.
Genome assembly	A singular (complete) or set of contigs after aligning and merging all sequence reads. Assemblies can be created *de novo* (relies only on sequence reads) or by mapping to a reference strain.
First-generation sequencing	Nucleotide sequencing that relied on either chain termination (Sanger) or cleavage (Maxam-Gilbert) methodology in single-tube reactions.
Second-generation sequencing	Nucleotide sequencing methods that sheared the genome and PCR amplified individual DNA fragments to massively parallelize sequencing and detect base identity by monitoring release of pyrophosphate (454), release of hydrogen (Ion Torrent), release of fluorescent reversible-terminator nucleotides (Illumina), or fluorescent ligated probes (SOLiD).
Third-generation sequencing	Nucleotide sequencing that relies on real-time single-molecule sequencing via monitoring of fluorescently labeled nucleotide incorporation (Pacific Biosciences) or ion current after DNA is fed through a channel (Oxford Nanopore).

Third-generation single-molecule sequencing, exemplified by Oxford Nanopore Technologies (ONT) and Pacific Biosciences (PacBio) technologies, produces much longer reads (typically 5 to 100 kbp, but there are recent reports of reads of >2 Mb [16]). Genome assemblies generated with long reads have fewer gaps and often span lengthy repeat regions, allowing the resolution of complex structural features, such as tandem repeats and nested insertions (17). Third-generation technologies have higher cost per base and higher per-base error rates than Illumina (5 to 15%), although improvements to chemistry and base calling algorithms are reducing the differential (18, 19). Error rates can in part be compensated by increasingly achievable higher read depths. For example, recent ONT studies generated >100× genome coverage and consensus error rates of <0.08% (20, 21). This level of error is adequate for most WGS-AST methods based on gene detection but may still be too high for many single-nucleotide polymorphism (SNP)-based methods. Hybrid assemblies (22) combine the accuracy of Illumina data and the gap-free assembly of ONT/PacBio and can achieve accuracies of >99.9%, but they rely on creating multiple libraries per sample, hence increasing cost and data management complexity.

The ONT technology has particular potential for WGS-AST because DNA sequence data become available within minutes of starting the sequencing run. A number of clinical gene-based ASTs for ONT have been piloted (see, e.g., references 21 and 23–25). At least two publications have demonstrated “streaming” AST, where the antibiotic resistance profile is updated in real time as the sequencing data are produced by the instrument (20, 26). Břinda et al. (26) used an indirect lineage-based method for predicting the resistance phenotype, which has the potential to mitigate some of the disadvantages of low-coverage ONT data, especially low SNP calling accuracy.

The outlook for clinical sequencing constantly shifts as existing technologies mature and become more cost-effective and new approaches emerge. Currently, Illumina is the dominant platform for WGS-AST. However, the approximate minimum cost of $80 per genome is still too high for routine use in clinical laboratories. Minimum costs are based on 1-week turnaround times and batching many samples together for efficiency. Obtaining results within <24 h for small numbers of samples is possible but comes at a price penalty. Further decreases in cost and turnaround times will be needed before sequencing will be economically viable for routine WGS-AST. New instrumentation, such as the ONT Flongle disposable flow cells, might allow quicker and less expensive sequencing of smaller numbers of strains. Emerging synthetic long-read technologies (e.g., 10× Genomics) that rely on the barcoding of long DNA fragments to associate the resulting short reads during assembly (27) may provide the combination of read length, accuracy, and reduced cost that opens the door for routine WGS-AST and eventually routine metagenomic-AST.

## WGS-AST BASED ON SEARCHING CATALOGS OF RESISTANCE LOCI

At its essence, WGS-AST attempts to estimate the phenotype that would have been ascertained if the strain were subjected to a gold standard culture-based antibiotic resistance test. The simplest approaches seek to classify the strain as either susceptible or resistant to specific antibiotics, as defined by CLSI or EUCAST guidelines (3). More complex models have further tried to predict the MIC of an antibiotic for the strain. The most straightforward approach is to use a “rules-based” classification based on the presence of one or more known antimicrobial resistance (AMR) genes or mutations (Tables 2 and 3). This requires cross-referencing the genome sequence against databases of antibiotic resistance determinants. Databases have been developed mostly from curation of the literature on molecular genetic studies that link antibiotic resistance phenotypes to genes (28). Multispecies databases include CARD (29, 30), ResFinder (31) and its companion PointFinder (32), ARG-ANNOT (33), ARDB (34), MEGARes (35), Resfams (36), RAST (37), and the Bacterial Antimicrobial Resistance Reference Gene Database (BARRGD; https://www.ncbi.nlm.nih.gov/bioproject/313047). In addition, there are databases developed for single organisms, such as Dream TB (38) and MUBII-TB-DB (39) for M. tuberculosis.

**TABLE 2:** Types of antibiotic resistance loci

Locus type	Description^*a*^
Gene	Presence of an intact protein-coding gene that confers resistance. For example, a strain that contains *blaZ* is inferred to be resistant to beta-lactams.
Plasmid/mobile element	Presence of a known drug resistance plasmid or mobile genetic cassette (e.g., SCC*mec* [114]) is used to infer that *S. aureus* is resistant to a beta-lactams.
Mutation	A particular SNP or SNV (which encompasses both SNPs and 1-bp indels) that is associated with resistance.
Allele	Nucleotide variant of gene caused by mutation. One sequence variant of a gene may be sensitive to a drug, while another allele may be associated with resistance.
Gene amplification	Increase in gene copy number due to homologous recombination. For example, a single gene in a genome may be sensitive to a drug, but a strain with two or more tandem repeats may be resistant.

a SCC*mec*, staphylococcal cassette chromosome *mec* element; SNV, single-nucleotide variant.

**TABLE 3:** Selected rule-based WGS-AST results^*a*^

Species	Antibiotic(s)	No. of genomes tested	Diversity/no. of STs	Primary database(s)	Software	Input data	Sensitivity (%)	Specificity (%)	Reference
E. coli	Amoxicillin-clavulanate	76	NR	Custom	Blastx, ClustalW	Assembly, FASTQ	100	100	Tyson et al. (71)
	Trimethoprim	48	19+ STs	ResFinder	ResFinder	Assembly or FASTQ	100	100	Zankari et al. (115)
	Gentamicin	74	NR	>100 loci	Blastn	Assembly	100	100	Stoesser et al. (70)
M. tuberculosis	Pyrazinamide	167	NR	PhyResSE	Stampy	FASTQ	88.9	100, 94.9 (with uncharacterized)	Pankhurst et al. (64)
	Isoniazid	693	Lineages 1–4	TBDReaMDB, MUBII-TB-DB minus phylogenetic SNPs	TB Profiler	FASTQ	92.8	100	Coll et al. (63)
	Moxifloxacin	13,424	NR	NR	NR	NR	88.2	90	Miotto et al. (74)
	Amikacin	667	7 clades	Custom	SAMtools, mpileup, Cortex	FASTQ	91.2, 88.1 (with uncharacterized)	99.4, 99.5 (with uncharacterized)	Walker et al. (116)
	Rifampin	1,565	NR	Hain, Cepheid, AID^*b*^ assays, literature	Mykrobe	FASTQ	90.8	99	Bradley et al. (5)
	Ethambutol	752	NR	Mykrobe	Mykrobe	FASTQ	100	98.5, 77.3 (with uncharacterized)	Quan et al. (72)
S. aureus	Fusidic acid	491	61 STs	Custom	Blastn, tblastn	Assembly	91	100	Gordon et al. (66)
	Vancomycin	NR	16 CCs	Custom	Blastn, mapping software	Assembly, FASTQ	100	100	Aanensen et al. (67)
	Mupirocin	340	25 CCs	Modified from Gordon et al. (66)	Mykrobe	FASTQ	100	100	Bradley et al. (5)
	Ciprofloxacin, clindamycin, erythromycin, fusidic acid, gentamicin, methicillin, mupirocin, penicillin, rifampin, tetracycline, trimethoprim, vancomycin	1,379	111 STs	Custom	Mykrobe, GeneFinder, Typewriter	FASTQ, Assembly	97	99	Mason et al. (42)
*Salmonella enterica* serovar Typhi	Chloramphenicol	332	ST1 and ST2	CARD, ResFinder, literature	GeneFinder	FASTQ	100	100	Day et al. (61)
Non-serovar Typhi *S. enterica*	Ceftriaxone	640	NR	Tyson et al. (71)	Blastx, ClustalW	Assembly, FASTQ	100	99.8	McDermott et al. (59)
	Ciprofloxacin	3,491	227 serovars	CARD, ResFinder	GeneFinder	FASTQ	99.28	99.97	Neuert et al. (60)
S. pneumoniae	Erythromycin	210	90 STs	SRST2	SRST2	FASTQ	100	100	Deng et al. (69)
Campylobacter jejuni, Campylobacter coli	Erythromycin	32/82	NR	Tyson et al. (71), ARDB, ResFinder	Blastx, ClustalW	Assembly, FASTQ	100	100	Zhao et al. (62)
Enterococcus faecalis, Enterococcus faecium	Kanamycin	50	12 STs 17 STs	ResFinder	ResFinder	Assembly or FASTQ	100	100	Zankari et al. (115)
Pseudomonas aeruginosa	Levofloxacin	390	175 STs	Custom	NR	Assembly	91.9	93.7	Kos et al. (68)
Klebsiella pneumoniae	Gentamicin	69	NR	Custom	Blastn	Assembly	96	100	Stoesser et al. (70)
Shigella sonnei	Ampicillin	341	NR	CARD, ResFinder	GeneFinder	FASTQ	100	100	Sadouki et al. (65)

a NR, not reported; ST, sequence type; CC, clonal complex.

b AID, Autoimmun Diagnostika GmbH.

Software tools for rules-based matching of antibiotic resistance catalogs operate on data produced at two points in the workflow for next-generation sequencing, raw sequence data and assembled contigs (Fig. 1). Each has tradeoffs in terms of speed of result and accuracy.

Detecting resistance in raw reads obviates the need for assembly and can therefore can reduce the time to result if the algorithms are efficient. However, false positives may be introduced because of sequencing errors present in individual reads or DNA contamination from other organisms. Setting minimum thresholds for the number of reads needed for a positive result can help overcome read error problems. Software tools use different strategies for processing raw reads. KmerResistance (40) matches k-mer subsequences of the resistance locus catalog against raw reads similarly split into the same length subsequence. SRST2 (41) and GeneFinder (42) use the efficient read alignment program Bowtie (43) to map genes to the read set as a first step before enumerating SNPs and gene matches. ARIBA (44) uses a partial *de novo* assembly after first recruiting individual reads that may map to target genes. The web tool Point-Finder (32) identifies known point mutations after mapping reads against reference genomes. Mykrobe (5) creates de Bruijn graphs of contigs from raw data and matches them against known genes; however, for speed, it omits generation of a consensus sequence (42).

Genome assembly can either be *de novo* or by mapping to a reference strain. *De novo* assembly usually produces more fragmented genome assemblies but avoids the biases of building the assembly on an existing reference template. For reference-based assembly, single nucleotide polymorphism (SNP) detection becomes less accurate the greater the distance between isolate and reference, but *de novo*-assembled DNA sequences are free from this bias. However, an antibiotic resistance gene may be missed when using a *de novo* assembly if it is split across multiple contigs. Multicopy genes associated with antibiotic resistance, such as rRNA, present a particular challenge. A mutation in just one or two copies is sometimes sufficient to impart a resistance phenotype (e.g., in the case of azithromycin resistance caused by mutations in Neisseria 23S rRNA [45]), yet assembly algorithms seek a consensus sequence; thus, important genetic variants within repeats could be missed if repeats “collapse” into a single copy (3, 46). Using longer reads as input for assembly can overcome the problem of collapsed repeats. Increasing sequence coverage should also minimize these types of errors, but more data come with increased cost and slower computation time. This can be partially addressed by downsampling coverage, as after about 100× Illumina genome coverage of Staphylococcus aureus genomes, adding more data to the assembly produced little extra benefit for assembly accuracy at the cost of significant declines in processing speed (47). Most catalog-based software programs that take assembled data as input (e.g., Typewriter [42], SSTAR [48], CARD RGI [30], ARG-ANNOT [33], ResFinder [31], and ABRicate [https://github.com/tseemanNAbricate]) use some form of BLAST alignment and results parsing, which usually takes a small fraction of the processor time used to construct the *de novo* assembly.

For many species and antibiotic resistance phenotypes, there is good concordance between what is known about the genetic basis of resistance and the resistance phenotype. Rules-based WGS-AST has been shown to have high sensitivity and specificity (>95%) for many phenotypes across several pathogen species (Table 3) (5, 32, 40, 42, 49–74), although with the caveat that studies varied widely in the number of strains tested and the within-species genetic diversity of the test set. However, there were some cases, e.g., levofloxacin resistance in P. aeruginosa (Table 3) (68), where sensitivity and specificity were below 95%. In an extensive survey of the genetic basis of resistance in M. tuberculosis, Miotto et al. (74) classified the predictive power of M. tuberculosis mutations into high, moderate, and minimal confidence. Therefore, rules-based approaches alone may not always be sufficient for accurate WGS-AST.

## MODEL-BASED ANTIBIOTIC RESISTANCE PREDICTION

Most rules-based methods make a number of (often unacknowledged) assumptions about the phenotypes they attempt to predict. These assumptions include (i) that either a single genetic locus is responsible for the phenotype, or, if multiple loci are present, that they do not interact in a complex manner (i.e., absence of epistasis [75–77]); (ii) that loci are highly penetrant and are not affected by the strain background; and (iii) that there is complete knowledge of the genetic basis of the phenotype. For a large number of cases, these assumptions do not completely hold. Some studies have attempted to capture uncertainty in the genetic basis of resistance and reduce overfitting using a variety of statistical modeling and machine learning (ML) approaches (Table 4) (45, 78–85). For simplicity, we have placed them together here under the term “model-based” prediction.

**TABLE 4:** Selected model-based WGS-AST results

Species	Antibiotic(s)	No. of genomes tested	Diversity^*a*^	Database^*b*^	ML algorithm	Input data	Sensitivity (%)	Specificity (%)	Overall accuracy (%)^*c*^	Reference
E. coli	Amoxicillin	329	7 STs	NA	Gradient-boosted trees	Pangenome, population structure matrix	90	95		Moradigaravand et al. (78)
	Ciprofloxacin	581	7 STs	NA	Gradient-boosted trees	Pangenome, population structure matrix, SNPs	81	99		Moradigaravand et al. (78)
	Gentamicin	564	7 STs	NA	Gradient-boosted trees	Pangenome, population structure matrix	87	99		Moradigaravand et al. (78)
	Trimethoprim	283	7 STs	NA	Gradient-boosted trees	Pangenome, population structure matrix	92	97		Moradigaravand et al. (78)
M. tuberculosis	Isoniazid	1,811 (80% train, 20% test)	7 clades	NA	Random Forest	Variants in 23 genes	97	94		Yang et al. (79)
	Rifampin	1,725 (80% train, 20% test)	7 clades	NA	Class-conditional Bernoulli mixture model	Variants in 23 genes	97	97		Yang et al. (79)
	Ethambutol	3,526 (80% train, 20% test)	5 genetic clusters	NA	Multitask wide and deep neural networks	Variants in 32 regions	91.9	90.3		Chen et al. (81)
	Pyrazinamide	3,147 (train), 567 (test)	5 genetic clusters	NA	Multitask wide and deep neural networks	Variants in 32 regions	75.2	90.1		Chen et al. (81)
	Kanamycin	162 (train), 18 (test)	NR	PATRIC, RAST	AdaBoost	Assembly			88.3 (F1)	Davis et al. (83)
S. pneumoniae	Beta-lactams (PEN, AMO, MER, TAX, CFT, CFX)^*d*^	2,528 (train), 1,781 (test)	403 STs (train), 299 STs (test)	NA	Random Forest	PBP sequences			>97 (±1 MIC dilution), >93 (category)	Li et al. (80)
	Beta-lactams	1,350 (train), 58 (test)	NR	PATRIC, RAST	AdaBoost	Assembly			87.6 (F1)	Davis et al. (83)
N. gonorrhoeae	Azithromycin	681	NR	NA	Linear regression	Variants in 20 regions	80, 99 (±1 MIC dilution)	83, 94 (±1 MIC dilution)	93 (±1 MIC dilution), 44 (category)	Eyre et al. (45)
	Ciprofloxacin	676	NR	NA	Linear regression	Variants in 20 regions	100	99	94 (±1 MIC dilution), 68 (category)	Eyre et al. (45)
K. pneumoniae	Ampicillin-sulbactam	1,668	>99 STs	PATRIC, RAST	XGBoost	Assembly			99 (F1, ±1 MIC dilution)	Nguyen et al. (117)
	Levofloxacin	1,668	>99 STs	PATRIC, RAST	XGBoost	Assembly			93 (F1)	Nguyen et al. (117)
	Meropenem	1,777	>99 STs	PATRIC, RAST	AdaBoost	Assembly			92 (F1)	Long et al. (118)
	Piperacillin-tazobactam	1,777	>99 STs	PATRIC, RAST	AdaBoost	Assembly			76 (F1)	Long et al. (118)
E. coli, *Enterobacter aerogenes*, K. pneumoniae	Ampicillin	78	NR	Resfams	Logistic regression	FASTA, alignments			97.4	Pesesky et al. (85)
	Chloramphenicol	78	NR	Resfams	Logistic regression	FASTA, alignments			89.7	Pesesky et al. (85)
S. aureus	Methicillin	99 (train), 11 (test)	NR	PATRIC, RAST	AdaBoost	Assembly			99.5 (F1)	Davis et al. (83)
	Vancomycin	75	12 STs	Custom	Random Forest	Assembly	73		81	Alam et al. (82)
Acinetobacter baumannii	Carbapenem	99 (train), 11 (test)	NR	PATRIC, RAST	AdaBoost	Assembly			95 (F1)	Davis et al. (83)
Non-serovar Typhi *S. enterica*	Ceftriaxone	5,278		PATRIC, RAST	XGBoost	Assembly			80, 95 (±1 MIC dilution)	Nguyen et al. (84)

a ST, sequence type; NR, not reported.

b NA, not applicable.

c F1, harmonic average of the precision (positive predictive value [PPV]) and recall (sensitivity) (84).

d PEN, penicillin; AMO, amoxicillin; MER, meropenem; TAX, ceftriaxone; CFT, cefotaxime; CFX, cefixime.

The most common strategy used is to train a classifier on a set of genomes with known phenotypes. The classifier can be asked to learn which SNPs, indels, or other genetic features are important for the phenotype *ab initio* or be given a set of features already known to be important based on existing databases or a combination of both. As not all AMR determinants contribute equally to the antibiotic resistance of a strain, noise in phenotype prediction can often be reduced and accuracy increased by weighting each locus using a machine learning model. Models can also be trained to take into account potential interactions between loci. The accuracy of the model is determined by predicting resistance in a second set of phenotyped genomes, ideally those from strains different from the training set. Models can be used to predict either sensitivity or resistance based on an accepted threshold or the MIC of the strain to the particular antibiotic. As for rules-based methods, data inputs can be reads, k-mers, and assembled contigs (Table 4). Time to result is highly variable and is dependent on factors, such as the number of features used and the complexity of the ML algorithm. Three examples, discussed briefly below, illustrate different model-based approaches to prediction and some of the advantages and pitfalls.

Yang et al. (79) examined the genomes of 1,839 M. tuberculosis strains resistant to eight drugs isolated in the UK, using reference mapping to identify mutations in 23 putative resistance genes identified in earlier experimental studies. Because of the lack of lateral gene transfer in M. tuberculosis, resistance arises primarily through mutations (in this case, SNPs). Seven ML models were built and compared to simple rules-based models, where the presence of a known mutation indicated resistance. Different subsets of mutations were also tested to determine the effect on performance. The best ML method for each drug increased sensitivity over the rules-based models by 2 to 24%, with the trade-off of minor losses in specificity due to strains labeled as “susceptible” containing mutations associated with resistance. However, it is possible that the phenotypes of these strains were determined incorrectly. The best model for each drug varied in both the ML algorithm used and the mutation subset used, indicating that there is no one-size-fits-all solution, and the model chosen for prediction can be optimized for the complexity of resistance phenotype and amount of *a priori* knowledge.

In an alternative approach to the multiple-locus problem, Eyre et al. (45) predicted Neisseria gonorrhoeae MICs to cefixime, penicillin, azithromycin, ciprofloxacin, and tetracycline using multivariate linear regression. Multiple genetic loci (SNPs, plasmids, and alleles of the *penA* gene) were already known to make contributions to resistance. The presence or absence of each candidate locus was ascertained in 681 N. gonorrhoeae genomes by mapping reads to a reference genome. Backwards selection (where each variable is removed until the information lost, as estimated by the Akaike information criterion score, is minimized) was used to reduce the number of loci in the model and limit overfitting. Overall, model-fitted MICs were within two doubling dilutions of the MIC for 98% of the strains. Using EUCAST cutoffs, the sensitivity of calling resistance was 98.7%, and the specificity was 98.3%.

The final example of successful implementation of model-based methods is the prediction of beta-lactam resistance in Streptococcus pneumoniae (80, 86). Most of the strain-specific variation in levels of resistance in S. pneumoniae was found to be driven by amino acid sequence variation in the transpeptidase domain of three penicillin binding proteins (PBPs). Proteins with similar sequence signatures in their transpeptidase domains were clustered into “PBP types.” There were high levels of horizontal transfer of genes in the S. pneumoniae species, but the strain genomic background outside the PBPs only contributed 1 to 6% of the variation in MIC (86); instead, the PBP type was the most important variable. In 4,309 Streptococcus genome sequences where the PBP type was ascertained from *de novo*-assembled data (80, 87), the MIC could be accurately predicted using both a rules-based model (“Mode MIC,” where the MIC was the most frequent in the closest known PBP type) and a machine learning (Random Forest) classifier using the amino acid sequence of the transpeptidase domains. Mode MIC and Random Forest false-positive rates were <3%, but critically, while the false-negative rates for the Random Forest classifier were low, the rates for Mode MIC were above 50% for some beta-lactams. The reason for the poor performance of the rule-based prediction was the misclassification of a subset of PBP types with significant sequence divergence from the training set. The good performance of the Random Forest classifier across previously unseen sequences was because the machine learning method discovered the previously unappreciated significance for resistance of a group of key amino acid residues in the transpeptidase domains supplied in the training set.

## CONSEQUENCES OF INCORRECT AST PREDICTION AND METHODS TO INCREASE ROBUSTNESS

Errors in the sensitivity and specificity of genome prediction of antimicrobial phenotypes, either false positives (phenotypically susceptible, WGS-prediction resistant) or false negatives (phenotypically resistant, WGS-prediction susceptible) have different consequences for treatment. False negatives are considered most concerning, as they can lead to inadequate treatment of a resistant infection, increasing morbidity and mortality. It is often preferable to reduce false negatives at the cost of increasing the false-positive rate (66), although false-positive results lead to inappropriate antibiotic use, potentially harming the patient and increasing the risk of resistance to last-line antibiotics.

How can the accuracy of genetic prediction be improved in the future? It is important in development of WGS-AST tools to have as large and diverse of a test and training strain set as possible. Most studies to date have used a convenience sample of strains based on accessible collections with limited geographic and temporal variability. Even if large numbers of strain genomes are obtained, accuracy statistics can be misleading if isolates from locally abundant clonal lineages are heavily overrepresented. In reporting the isolate collection used for WGS-AST development and testing, we need to develop accepted statistics for the assessment of genetic diversity. These could be pairwise average nucleotide identity, number of multilocus sequence type (MLST)/cgMLST, a sequence entropy measure, or the percentage of known species pangenome represented. Epistatic effects could explain the reduced accuracy of WGS-AST across diverse strain backgrounds, especially those not included in test sets. The relatively few studies on epistasis and antibiotic resistance suggest that few generalizations can be made across species and phenotypes. Knopp and Andersson (77) found little variation in phenotypic expression for 13 resistance mutations across 10 strains of Salmonella enterica and Escherichia coli (even when as many as four mutations were combined in one background). However, there are reports of epistatic effects both between resistance mutations and between the resistance mutation and the strain genetic background in Pseudomonas aeruginosa and other species (75, 88, 89). Extensive empirical testing will be needed to understand how resistance phenotype expression varies across diverse strain backgrounds in each species. New statistical methods coupled with very large pathogen genomic data sets can also lead to the discovery of novel epistatic interactions (90).

The finding that rules-based methods for predicting beta-lactam MICs from PBP type performed poorly on previously unseen strain types (80), discussed above, is an example of how incomplete knowledge impacts WGS-AST. We do not yet understand the genetic basis of resistance for some antibiotics. For others, even where there are well-defined canonical mutations or genes, it is becoming apparent that there are a large number of rare genetic events that could lead to resistance. Guérillot et al. (91) found through deep sequencing of lab-selected colony pools that while eight *rpoB* mutations were responsible for 93% of the rifampin resistance in S. aureus clinical strains (92), the remaining 8% were caused by least 72 rarer mutations (91). Wistrand-Yuen et al. (93) showed that selection for streptomycin resistance in Salmonella enterica at sub-MIC levels resulted in strains with an array of novel low-effect mutations different from classic high-effect drug resistance. Potentially even harder to predict based on sequence alone are resistance phenotypes caused by rare genetic events, such as insertion sequence movement into or upstream of open reading frames (94, 95).

In the face of this genetic complexity, how can WGS-AST continue to improve? It is unrealistic to expect 100% accuracy for every drug. We may be able to establish a stable level of uncertainty for each phenotype and learn to accept that some are more genetically plastic than others. Improvements may also be possible by incorporating functional prediction algorithms for mutations in resistance genes where the functional effect is untested. At its simplest, this could be taking into account frameshift mutations that, for example, in a *blaZ* gene would be predicted to inactivate translation of the beta-lactamase and yield a phenotypically susceptible strain. More sophisticated metrics include delta-bitscore, which uses hidden Markov models to identify functional divergence in orthologous genes and is an improvement on the previous use of the ratio of nonsynonymous to synonymous evolutionary changes (*dN*/*dS*) (used to indicate positive selection), which was prone to false positives (96). S. aureus resistance to trimethoprim has been accurately predicted based on the free energy state of the dihydrofolate reductase amino acid sequence (97), suggesting an avenue for sophisticated structural prediction studies. One straightforward way to increase robustness of WGS-AST prediction is to combine predictions of multiple tools that examine different facets of the WGS data set (i.e., k-mers and assembled contigs) (42).

## THE PHENOTYPE PROBLEM

As mentioned earlier, WGS-AST uses culture-based antibiotic phenotypes as the gold standard, but culture-based methods are not themselves free of uncertainty. Culture-based assays for drugs in some species have been shown to be unreliable, for example, ethambutol, pyrazinamide, and rifampin in M. tuberculosis (98–101). Even for drug resistances that can be tested reliably, there is evidence to suggest that the error in culture-based AST between different reference laboratories is greater than the inaccuracy of WGS-AST (45, 57, 67). Errors may arise from subtle operator-specific biases or variations between individual automated instruments that perform rapid organismal identification and susceptibility testing. Additionally, unlike standard research laboratory experimentation, it is generally not practical to perform replicate MIC tests in clinical laboratories.

Culture-based AST measures resistance in a highly constrained setting that does not reflect the variance of expression of the phenotype, especially during human infections. Many culture-based MICs are not robust to changes in growth medium, bacterial cell density, or temperature. Antimicrobial susceptibility may be altered due to the formation of biofilms and/or the synergistic or antagonistic interactions with other bacterial species (102–104). Resistance gene expression can be induced to higher levels *in vivo* than *in vitro* (105), small resistant subpopulations (heteroresistant strains) or mixed infections can quickly exhibit phenotypic resistance when exposed to selection, and persisters (106), which are not detected by AST, can survive treatment and cause recurrent infections. This underlies the fundamental issue of using MIC breakpoints to define resistance. Breakpoints are not based on genetic correlates of resistance but on factors, such as distribution of MICs, pharmacokinetics of the drug, and clinical treatment experience, among others. Consequently, there is considerable debate on how to set breakpoints, which can vary across countries and organizations (107). Breakpoints are often revised due to new epidemiological data, but adoption can sometimes be slow, and changes can serve to confound retrospective meta-analyses of antibiotic resistance trends and mechanisms defined in the literature.

Despite culture-based AST being an imperfect gold standard for WGS-AST, it is unlikely that it will soon be replaced. However, due to a revolution in NAAT-based typing, clinical laboratories are culturing many fewer isolates, especially hard-to-grow species, such as Legionella spp. and M. tuberculosis, or sexually transmitted diseases, such as those caused by *Neisseria* spp., which need a fast turnaround time for diagnosis. As sequencing becomes less expensive and more accurate, metagenomic-AST (Fig. 1) may render culturing of isolates unnecessary for routine treatment. However, as bacterial pathogens continue to evolve, it is possible that new resistance loci will emerge or the relationship between known AMR determinants and phenotypic resistance will change due to epistatic interactions with novel emerging strain backgrounds. Therefore, even when phenotypic testing is no longer used as a primary screen, it should continue to be used as quality control step to ensure that contemporary clinical strains have not drifted away from predicted phenotypes. Large multisite surveys that integrate rational strain sampling and centralized gold standard AST, such as the recent European N. gonorrhoeae study (57), will be needed to continually update and challenge existing WGS-AST methods.

## CONCLUDING REMARKS

The adoption of routine use of whole-genome sequencing in clinical microbiology would be a revolution in clinical medicine. While this review only discusses bacterial pathogens, there is also progress being made to use WGS to predict drug resistance in viruses, fungi, and eukaryotic parasites (108–110). The major barriers to adoption for routine use stem from technological limitations that make the process currently too slow and expensive and the need to demonstrate the advantages of a new approach to diagnosis for skeptical end-users in clinical microbiology laboratories (Table 5). Along with the development of instrumentation, there is need for efficient data management of WGS data, user-friendly pipelines that do not require specialized bioinformaticians for use, and rapidly interpretable results (111). As has been covered in more depth in other articles (3, 4, 112), standardization of software, sequence data, genomic test sets and phenotypes, and data storage are all needed to transform what is now a research enterprise into a robust clinical tool acceptable to both clinical microbiologists and regulatory agencies.

**TABLE 5:** Some outstanding questions

Question
At what price and turnaround time will WGS-AST replace culture-based sequencing for routine use in clinical microbiology labs?
How do we interpret the presence of an antimicrobial resistance determinant gene if the susceptibility of the strain is below the MIC?
Can genome prediction be used to detect heteroresistance? Or to detect polygenic phenotypes?
How important is epistasis in determining the resistance to different classes of antibiotics?
Can gene amplification as a mechanism of resistance be accurately determined from WGS data?
How efficiently can WGS-AST prediction software be ported to metagenomic-AST data?
